# Crystal structure of a tri­fluoro­methyl benzoato quadruple-bonded dimolybdenum complex

**DOI:** 10.1107/S205698902200010X

**Published:** 2022-01-07

**Authors:** Elisabeth Aigeldinger, Lilliana Brandao, Troy Powell, Alaina C. Hartnett, Rui Sun, Dilek K. Dogutan, Shao-Liang Zheng

**Affiliations:** a Department of Chemistry and Chemical Biology, Harvard University, Cambridge, MA 02138, USA

**Keywords:** crystal structure, quadruple bond, molybdenum, delta bond

## Abstract

The study of quadruple bonds between transition metals, in particular those of dimolybdenum, has revealed much about the two-electron bond. The solid-state structure of the quadruple-bonded dimolybdenum(II) complex tetra­kis­[μ-4-(tri­fluoro­methyl)­benzoato-κ^2^ *O*:*O*′]bis[(tetra­hydro­furan-κ*O*)molybdenum(II)] 0.762-pentane 0.238-tetra­hydro­furan solvate, [Mo_2_(*p*-O_2_CC_6_H_4_CF_3_)_4_·2THF]·0.762C_5_H_12_·0.238C_4_H_8_O or [Mo_2_(C_8_H_4_F_3_O_2_)_4_(C_4_H_8_O)_2_]·0.762C_5_H_12_·0.238C_4_H_8_O is reported. The complex crystallizes within a triclinic cell and low symmetry (*P*  ) results from the inter­calated penta­ne/THF solvent mol­ecules. The paddlewheel structure at 100 K has inversion symmetry and comprises four bridging carboxyl­ate ligands encases the Mo_2_(II,II) core that is characterized by two axially coordinated THF mol­ecules and an Mo—Mo distance of 2.1098 (7) Å.

## Abstract 2

The quadruple-bond complex, [Mo_2_(*p*-O_2_CC_6_H_4_CF_3_)_4_·2THF], crystallizes in the triclinic space group *P* 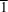 with inter­calated penta­ne/THF lattice solvent mol­ecules. The electron-withdrawing group on the paddlewheel carboxyl­ate together with the axial THF mol­ecules lead to a slight lengthening of the metal–metal bond, as predicted by Cotton.

## Chemical context

The σ^2^π^4^δ^2^ quadruple bond has contributed prominently to the elucidation of the single most distinguishing feature of the discipline of chemistry – the two-electron bond (Lewis, 1916 ▸). As originally defined with the inception of valence and mol­ecular orbital bonding models (Heitler & London, 1927 ▸; Pauling, 1928 ▸; Lennard-Jones, 1929 ▸; Mulliken, 1932 ▸; James & Coolidge, 1933 ▸; Coulson & Fischer, 1949 ▸), the two-electron bond forms from pairing two electrons in two orbitals. Remarkably, the four states that characterize the two-electron bond remained undefined experimentally for over 60 years owing to the dissociative nature of the σ and π anti­bonding orbitals. This experimental challenge was overcome with the two-electron δ bond of quadruple-bonded metal–metal complexes. Anchored by a σ^2^π^4^ framework and sterically locking ligands, the four states of the two-electron bond,^1^δδ, ^3^δδ*, ^1^δδ* and ^1^δ*δ*, were experimentally defined for dimol­yb­denum quadruple-bond complexes (Engebretson *et al.* 1994 ▸, 1999 ▸; Cotton & Nocera, 2000 ▸). Within the group of dimolybdenum quadruple-bond complexes, the tetra­acetates are exemplars. The initial structure of Mo_2_(O_2_CCH_3_)_4_ by Lawton & Mason (1965 ▸) established the existence of the quadruple bond in the now familiar paddlewheel arrangement of acetates. The dimolybdenum bond distance of 2.11 Å in this structure was subsequently refined nearly a decade later to be 2.0934 (8) (Cotton *et al.*, 1974 ▸). Intriguingly, many subsequent structures have shown that the inductive effect of the *R* group on the carb­oxy­lic acid does not perturb the Mo—Mo bond distance, indicating the robustness of the two-electron bond within a quadruple-bond architecture. It has been postulated that the strength of the Mo—Mo quadruple bond may be perturbed, but only in cases where *R* is a strong electron-withdrawing group and there is a good axial donor ligand (Cotton *et al.*, 1978 ▸). To add further to an understanding of Mo_2_(II,II) quadruple bond distances, we examined a dimolybdenum core ligated by tri­fluoro­meth­ylbenzoate with THF axial donor ligands. We now report the synthesis and X-ray crystal structure of tetra­kis­(μ-4-tri­fluoro­methyl­benzoato-κ^2^ *O*:*O*′)dimolybdenum(II) 0.762-pentane 0.238-tetra­hydro­furan solvate [Mo_2_(*p*-O_2_CC_6_H_4_CF_3_)_4_·2THF]·0.762C_5_H_12_·0.238C_4_H_8_O. The presence of the CF_3_ electron-withdrawing group on the bridging benzoate ligands, together with the donor THF axial ligands, results in a slightly elongated metal–metal bond distance as compared to its benzoate congener, Mo_2_(O_2_CC_6_H_5_)_4_. 
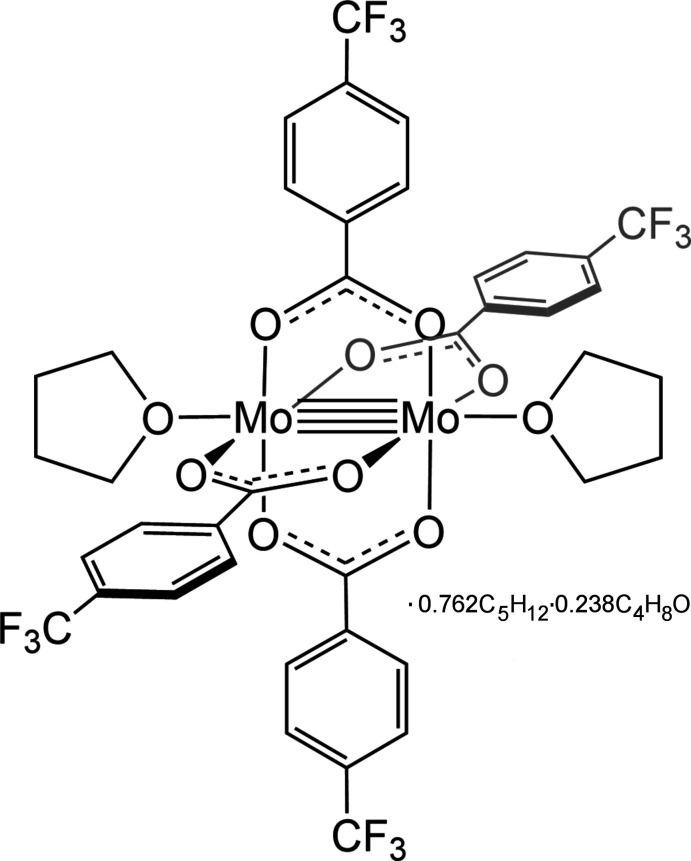


## Structural commentary

**Figure 1:**
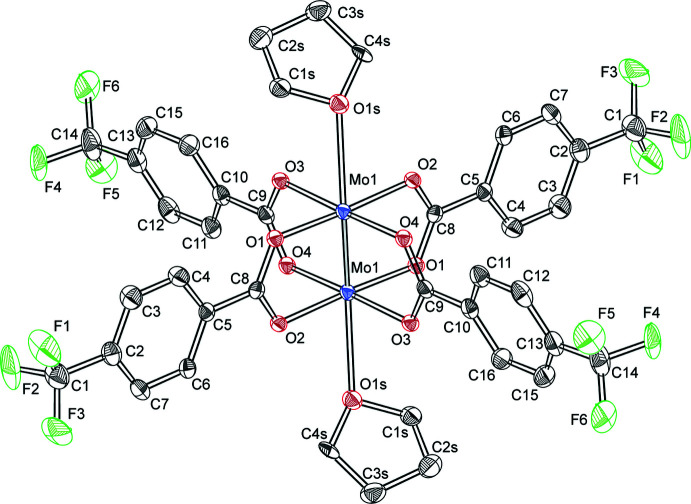
Ellipsoid plot of the dimolybdenum complex. The CF_3_ groups are rotationally disordered, therefore the highest occupancy positions are shown for each atom. Hydrogen atoms and unbound solvent are omitted for clarity.

**Figure 2:**
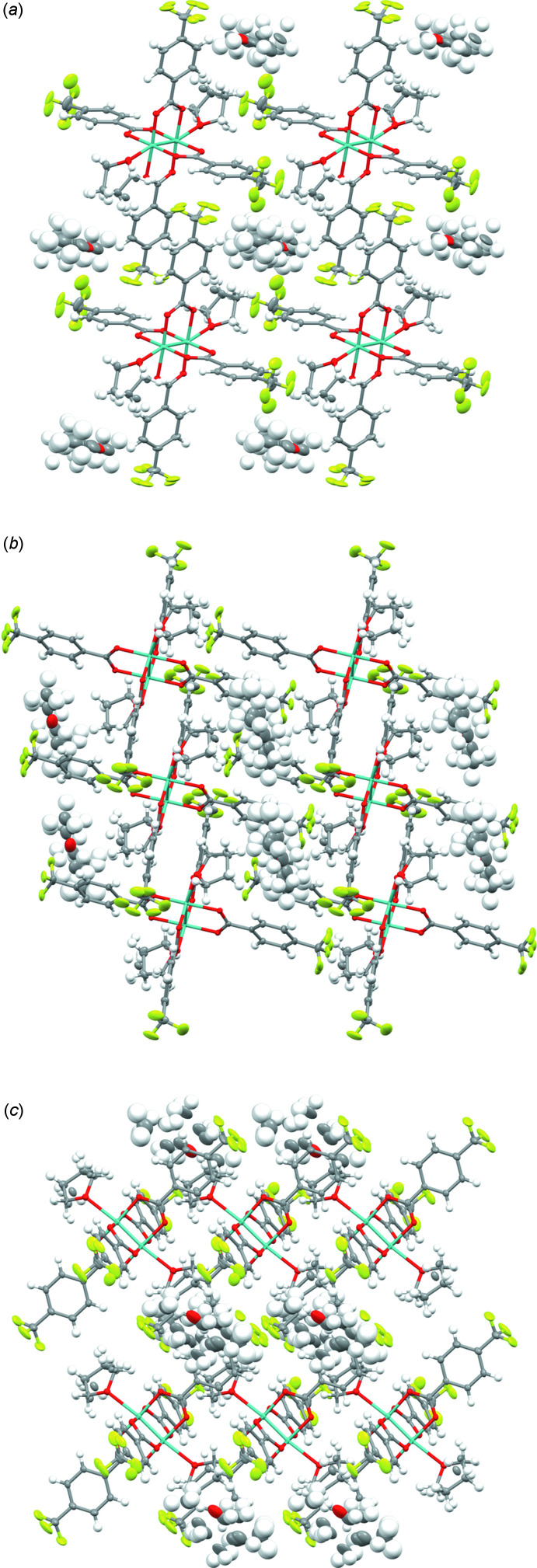
Crystal packing of the dimolybdenum complex shown along (*a*) the *a*-axis, (*b*) the *b*-axis and (*c*) the *c*-axis. The crystal has triclinic (*P* 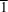 ) symmetry. Pentane and THF solvent mol­ecules are present in the structure. Color scheme: C (gray), O (red), H (white), F (green), Mo (teal).

The dimolybdenum complex, [Mo_2_(*p*-O_2_CC_6_H_4_CF_3_)_4_·2THF] (Fig. 1 ▸), was characterized by using single-crystal X-ray diffraction. Half of the mol­ecule (Fig. 1 ▸) resides in the asymmetric unit, with the complete mol­ecule generated by inversion about the quadruple-bond inversion center. The fluorine atoms of the tri­fluoro­methyl groups are rotationally disordered and the highest occupancy positions are shown in Fig. 1 ▸. The crystallization solvents, THF and pentane, are disordered (0.238:0.762) (Fig. 2 ▸).

Selected bond metrics for Mo_2_(*p*-O_2_CC_6_H_4_CF_3_)_4_·2THF are listed in Table 1 ▸. Complete lists of the structural metrics for the compound are presented in the Supporting information. The Mo—Mo bond length is 2.1098 (7) Å. Whereas the bond distance is within the typical range of dimolybdenum quadruple bond lengths of 2.06–2.17Å (Cotton *et al.*, 2002 ▸), it is slightly longer than what is observed for dimolybdenum cores bridged by carboxyl­ates. As a comparison, the dimolybdenum bond distance in the Mo_2_(O_2_CC_6_H_5_)_4_ congener, is 2.096 (1) Å. Thus, with the addition of a CF_3_ group in the 4-position of benzoate, the Mo—Mo bond length increases by 0.014 (2) Å. A similar trend is observed for the bond distances in the primary coordination sphere. The minimum Mo—O bond distance decreases by 0.008 (5) Å, and the maximum Mo—O bond distance decreases by 0.011 (5) Å as compared to Mo_2_(O_2_CC_6_H_5_)_4_. The most significant decrease in bond metrics is observed for the Mo—O1*S* axial ligand distance, which results in a decrease of 0.033 (4) Å for the axial coordinated oxygen atom of THF as compared to the axially coordinated oxygen in Mo_2_(O_2_CC_6_H_5_)_4_. However, we note for this compound that the oxygen is provided from a carboxyl­ate ligand of a neighboring mol­ecule as opposed to an axially coordinated solvent mol­ecule. Consequently, as proposed by Cotton (Cotton *et al.*, 1978 ▸), the presence of ligands about the dimolybdenum center that are electron withdrawing and donating in the axial position is needed to perturb the overall bonding within a quadruple-bond framework. To this point, the metrics of [Mo_2_(*p*-O_2_CC_6_H_4_CF_3_)_4_·THF] are indistinguishable from those of Mo_2_(O_2_CC_6_F_5_)_4_·THF (Han, 2011 ▸). The electron-withdrawing nature of the fluoro-substituted benzoates is established by their p*K*_a_s as compared to that of benzoate (p*K*_a_ = 1.75, 3.77 and 4.20 for C_6_F_5_COOH, *p*-CF_3_ C_6_H_4_COOH and C_6_H_5_COOH, respectively; Rumble, 2021 ▸; Boiadjiev & Lightner, 1999 ▸). That an electron-withdrawing group alone is insufficient to perturb the dimolybdenum bond distance is indicated by a comparison of the structures for Mo_2_(O_2_CCH_3_)_4_ and Mo_2_(O_2_CCF_3_)_4_. The *d*(Mo—Mo) of 2.0934 (8) and 2.090 (4) Å for Mo_2_(O_2_CCH_3_)_4_ and Mo_2_(O_2_CCF_3_)_4_, respectively (Cotton & Norman, 1971 ▸; Cotton *et al.*, 1974 ▸), are indistinguishable despite a significant difference in electron-withdrawing properties [p*K*_a_(CH_3_COOH) = 4.76, p*K*_a_(CF_3_COOH) = 0.52; Rumble, 2021 ▸]. Thus, a donor ligand is needed in addition to electron-withdrawing carboxyl­ate equatorial ligands to observe a difference in the dimolybdenum quadruple bond.

## Supra­molecular features

The structure was solved in the triclinic space group *P* 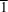 with a half of an Mo-dimer per asymmetric unit and one full mol­ecule per unit cell (Fig. 2 ▸). The low symmetry arises from the presence of disordered THF/pentane solvent mol­ecules embedded within a solvent channel arising from the crystal packing. The disordered solvents are situated in the body-center of eight [Mo_2_(*p*-O_2_CC_6_H_4_CF_3_)_4_·THF] complexes with two THF mol­ecules skewed towards the pentane; the next nearest neighbors are a series of four tri­fluoro­methyl groups from distinct [Mo_2_(*p*-O_2_CC_6_H_4_CF_3_)_4_·THF] complexes. These four tri­fluoro­methyl groups are oriented tangentially to the solvent channel (Fig. 2 ▸ *b*) along the *b*-axis direction with a volume of 162 Å^3^ for one void volume within the unit cell according to established methods for determining solvent-accessible voids (van der Sluis & Spek, 1990 ▸). The adjacent pairs of symmetry-related benzene rings (C10–C16) in the *p*-O_2_CC_6_H_4_CF_3_ ligands inter­act through aromatic π–π stacking inter­actions with a face-to-face distance of 3.7856 (9) Å (Fig. 2 ▸ *b*) and form a one-dimensional chain. In addition, the tri­fluoro­methyl group of a *p*-O_2_CC_6_H_4_CF_3_ ligand (for C10–C16 and F4–F6) is perpendicular to the aromatic plane of a neighboring *p*-O_2_CC_6_H_4_CF_3_ ligand (C1–C7 and F1–F3) with weak C—F⋯π inter­actions (Kawahara *et al.*, 2004 ▸) [the distances between the F atoms and the C2–C8 plane are 3.024 (2)–3.430 (1) Å]. The coordinated THF mol­ecules also have weak C—H⋯F inter­actions (D’Oria & Novoa, 2008 ▸) with the tri­fluoro­methyl group of the *p*-O_2_CC_6_H_4_CF_3_ ligands [the C—H⋯F distances are 2.568 (1)–3.045 (1) Å].

## Database survey

In a search of the Cambridge Structural Database (WebCSD, accessed 17 December 2021; Groom *et al.*, 2016 ▸), the CSD search fragment, C_32_H_16_F_12_Mo_2_O_8_, for Mo_2_(O_2_CC_6_H_4_CF_3_)_4_ yielded no hits in the database and the CSD search fragment, C_40_H_32_F_12_Mo_2_O_10_, for [Mo_2_(*p*-O_2_CC_6_H_4_CF_3_)_4_·THF] also yielded no hits. The CSD reference code for Mo_2_(O_2_CC_6_F_5_)_4_·THF (Han, 2011 ▸) is AYODOI, for Mo_2_(O_2_CC_6_H_5_)_4_ (Cotton *et al.*, 1978 ▸) is MOBZOA, for Mo_2_(O_2_CCH_3_)_4_ (Cotton *et al.*, 1974 ▸) is MOLACE01, and for Mo_2_(O_2_CCF_3_)_4_ (Cotton & Norman, 1971 ▸) is TFACMO.

## Purification and crystallization

The overall synthetic scheme is shown in the reaction scheme. Molybdenum hexa­carbonyl, 4-(tri­fluoro­meth­yl) benzoic acid, THF, and 1,2-di­chloro­benzene were purchased from Sigma-Aldrich. Mo(CO)_6_ and 4-(tri­fluoro­meth­yl)benzoic acid were combined in a flask with THF and anhydrous 1,2-di­chloro­benzene. The reaction was heated under reflux for 24 h at 413 K under nitro­gen (Pence *et al.*, 1999 ▸). The reaction mixture was cooled, the solution was filtered and the collected residue was washed with di­chloro­methane and hexa­nes. 
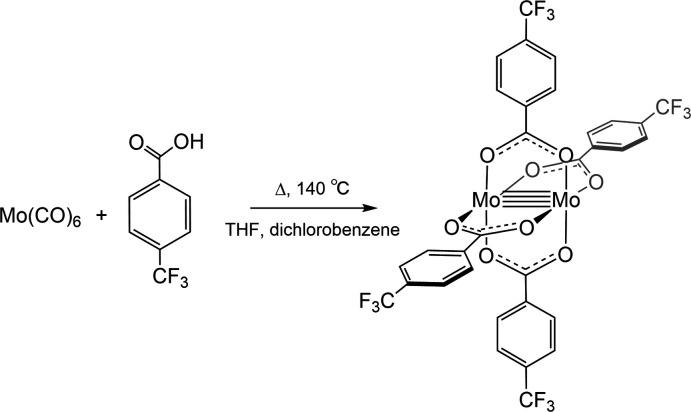


The crystallization was set up in a glove box. The crude product was dissolved in THF and recrystallized by vapor diffusion of pentane using a 6 by 50 mm borosilicate glass crystallization tube housed within a 20 mL glass vial. The assembly was allowed to stand at 238 K for 24 days. Orange rectangular crystals were observed and harvested for X-ray diffraction analysis.

## Refinement

Crystal data, data collection and structure refinement details are included in Table 2 ▸. Hydrogen atoms on C atoms were placed at idealized positions and refined using a riding model. The isotropic displacement parameters of all hydrogen atoms were fixed to 1.2 times the atoms to which they are linked (1.5 times for methyl groups). Rotational and positional disorder for one tri­fluoro­methyl substituent containing C1 and C13 was modeled. The overlapping solvent mol­ecules (assigned as THF and pentane based on solvent crystallization conditions and apparent arrangement of electron-density peaks) were disordered adjacent to an inversion center (special position). The restraints on bond lengths and constraints of the atomic displacement parameters on each pair of disorder fragments (SADI/SAME and EADP instructions of *SHELXL2014*) as well as the restraints of the atomic displacement parameters (SIMU/RIGU instructions of *SHELXL2014*) were applied for the disorder refinement (Zheng *et al.*, 2008 ▸). Crystallographic refinement details, including disorder modeling and the software employed, are given in the crystallographic information file (*.cif). To stabilize the refinement model, 713 restraints (SADI/SAME and RIGU/SIMU) were applied to accommodate the disordered tri­fluoro­methyl group, the coordinated THF mol­ecules, as well as the THF/pentane solvent mol­ecules in the channel as detailed by Müller *et al.* (2006 ▸) to furnish a data+restraint-to-parameter ratio of 9.75. This ratio increases to 11.6 if the disordered THF/pentane solvent mol­ecules in the channel are squeezed out of the structure.

## Supplementary Material

Crystal structure: contains datablock(s) I. DOI: 10.1107/S205698902200010X/mw2183sup1.cif

Structure factors: contains datablock(s) I. DOI: 10.1107/S205698902200010X/mw2183Isup2.hkl

CCDC reference: 2132473

Additional supporting information:  crystallographic information; 3D view; checkCIF report

## Figures and Tables

**Table 1:** Selected geometric parameters (Å, °)

Mo1—O1	2.0996 (17)	Mo1—Mo1^i^	2.1098 (7)
Mo1—O4	2.1030 (17)	Mo1—O3^i^	2.1204 (17)
Mo1—O2^i^	2.1076 (17)	Mo1—O1*S*	2.5422 (19)
			
O1—Mo1—Mo1^i^	93.20 (5)	O2^i^—Mo1—Mo1^i^	90.10 (5)
O4—Mo1—Mo1^i^	92.37 (5)	Mo1^i^—Mo1—O3^i^	90.84 (5)

Symmetry code: (i) -x, -y+1, -z+1.

**Table 2:** Experimental details

Crystal data	
Chemical formula	[Mo_2_(C_8_H_4_F_3_O_2_)_4_(C_4_H_8_O)_2_]·0.762C_5_H_12_·0.238C_4_H_8_O
*M*_r_	1164.68
Crystal system, space group	Triclinic, *P*\overline{1}
Temperature (K)	100
*a*, *b*, *c* (Å)	7.7687 (17), 12.099 (5), 12.572 (2)
α, β, γ (°)	85.843 (13), 81.208 (8), 83.107 (16)
*V* (Å^3^)	1157.6 (6)
*Z*	1
Radiation type	Mo *K*α
μ (mm^−1^)	0.65
Crystal size (mm)	0.30 × 0.13 × 0.06

Data collection	
Diffractometer	Bruker D8 goniometer with Photon 100 CMOS detector
Absorption correction	Multi-scan (*SADABS*; Krause *et al.*, 2015 ▸)
*T*_min_, *T*_max_	0.701, 0.745
No. of measured, independent and observed [*I* > 2σ(*I*)] reflections	39433, 4094, 3814
*R*_int_	0.033
(sin θ/λ)_max_ (Å^−1^)	0.597

Refinement	
*R*[*F* ^2^ > 2σ(*F* ^2^)], *wR*(*F* ^2^), *S*	0.028, 0.064, 1.12
No. of reflections	4094
No. of parameters	493
No. of restraints	713
H-atom treatment	H-atom parameters constrained
Δρ_max_, Δρ_min_ (e Å^−3^)	0.67, −0.39

Computer programs: *APEX2* and *SAINT* (Bruker, 2015 ▸), *SHELXT2014* (Sheldrick, 2015*a* ▸), *SHELXL2014* (Sheldrick, 2015*b* ▸), *SHELXTL* (Sheldrick, 2008 ▸), and *PLATON* (Spek, 2020 ▸).

## supplementary crystallographic information

### Crystal data

**Table:**

[Mo_2_(C_8_H_4_F_3_O_2_)_4_(C_4_H_8_O)_2_]·0.762C_5_H_12_·0.238C_4_H_8_O	*Z* = 1
*M**_r_* = 1164.68	*F*(000) = 586
Triclinic, *P*1	*D*_x_ = 1.671 Mg m^−^^3^
*a* = 7.7687 (17) Å	Mo *K*α radiation, λ = 0.71073 Å
*b* = 12.099 (5) Å	Cell parameters from 9835 reflections
*c* = 12.572 (2) Å	θ = 2.5–27.2°
α = 85.843 (13)°	µ = 0.65 mm^−^^1^
β = 81.208 (8)°	*T* = 100 K
γ = 83.107 (16)°	Block, orange
*V* = 1157.6 (6) Å^3^	0.30 × 0.13 × 0.06 mm

### Data collection

**Table:**

Bruker D8 goniometer with Photon 100 CMOS detector diffractometer	3814 reflections with *I* > 2σ(*I*)
Radiation source: IµS microfocus tube	*R*_int_ = 0.033
ω and phi scans	θ_max_ = 25.1°, θ_min_ = 2.7°
Absorption correction: multi-scan (SADABS; Krause *et al.*, 2015)	*h* = −9→9
*T*_min_ = 0.701, *T*_max_ = 0.745	*k* = −14→14
39433 measured reflections	*l* = −14→14
4094 independent reflections	

### Refinement

**Table:**

Refinement on *F*^2^	Primary atom site location: dual
Least-squares matrix: full	Hydrogen site location: inferred from neighbouring sites
*R*[*F*^2^ > 2σ(*F*^2^)] = 0.028	H-atom parameters constrained
*wR*(*F*^2^) = 0.064	*w* = 1/[σ^2^(*F*_o_^2^) + (0.0191*P*)^2^ + 2.0296*P*] where *P* = (*F*_o_^2^ + 2*F*_c_^2^)/3
*S* = 1.12	(Δ/σ)_max_ < 0.001
4094 reflections	Δρ_max_ = 0.67 e Å^−^^3^
493 parameters	Δρ_min_ = −0.39 e Å^−^^3^
713 restraints	

### Special details

**Table:**

Experimental. A single orange plate (0.297 mm × 0.132 mm × 0.056 mm) was chosen for single crystal X-ray diffraction using a Bruker three-circle platform goniometer equipped with an Photon100 CMOS detector. Data were collected as a series of φ and/or ω scans. Data integration down to 0.84 Å resolution was carried out using SAINT V8.37A with reflection spot size optimization. Absorption corrections were made with the program SADABS 2016/2 (Krause *et al.*, 2015). Space group assignments were determined by examination of systematic absences, *E*-statistics, and successive refinement of the structures. The structure was solved by the Intrinsic Phasing methods and refined by least squares methods also using SHELXT-2014 and SHELXL-2014 with the OLEX 2 (Dolomanov *et al.*, 2019) interface. The program PLATON (Spek, 2020) was employed to confirm the absence of higher symmetry space groups. All non-H atoms, including the disorder fragment, were located in difference Fourier maps, and then refined anisotropically. Outlier reflections were omitted from refinement when appropriate.
Geometry. All esds (except the esd in the dihedral angle between two l.s. planes) are estimated using the full covariance matrix. The cell esds are taken into account individually in the estimation of esds in distances, angles and torsion angles; correlations between esds in cell parameters are only used when they are defined by crystal symmetry. An approximate (isotropic) treatment of cell esds is used for estimating esds involving l.s. planes.
Refinement. Refinement of F^2^ against ALL reflections. The weighted R-factor wR and goodness of fit S are based on F^2^, conventional R-factors R are based on F, with F set to zero for negative F^2^. The threshold expression of F^2^ > 2sigma(F^2^) is used only for calculating R-factors(gt) etc. and is not relevant to the choice of reflections for refinement. R-factors based on F^2^ are statistically about twice as large as those based on F, and R- factors based on all data will be even larger. All non-H atoms, as well as the disordered atoms were located in difference-Fourier maps, and then refined anisotropically. The restraints on bond lengths and constraints of the atomic displacement parameters on each pair of disorder fragments (SADI/SAME and EADP instructions of SHELXL-2014) as well as the restraints of the atomic displacement parameters (SIMU/RIGU instructions of SHELXL- 2014), if necessary, have been applied for the disorder refinement.

### Fractional atomic coordinates and isotropic or equivalent isotropic displacement parameters (Å^2^)

**Table:**

	*x*	*y*	*z*	*U*_iso_*/*U*_eq_	Occ. (<1)
Mo1	−0.08669 (3)	0.43800 (2)	0.51162 (2)	0.01390 (8)	
C1	0.8554 (11)	0.0198 (7)	0.3139 (9)	0.0362 (17)	0.796 (10)
F1	0.8182 (10)	−0.0767 (5)	0.2806 (5)	0.0615 (15)	0.796 (10)
F2	0.9308 (6)	−0.0074 (4)	0.4035 (4)	0.0685 (14)	0.796 (10)
F3	0.9798 (5)	0.0542 (3)	0.2420 (4)	0.0629 (14)	0.796 (10)
C1A	0.840 (5)	0.013 (4)	0.301 (5)	0.054 (5)	0.204 (10)
F1A	0.817 (4)	−0.083 (2)	0.327 (2)	0.066 (5)	0.204 (10)
F2A	0.9862 (16)	0.0311 (12)	0.3439 (19)	0.059 (4)	0.204 (10)
F3A	0.908 (2)	0.0366 (15)	0.1933 (16)	0.080 (5)	0.204 (10)
C14	−0.273 (2)	0.6434 (15)	−0.1264 (11)	0.044 (4)	0.38 (3)
F4	−0.421 (2)	0.7128 (18)	−0.1227 (17)	0.052 (4)	0.38 (3)
F5	−0.303 (3)	0.5510 (13)	−0.1700 (15)	0.055 (3)	0.38 (3)
F6	−0.1534 (18)	0.692 (2)	−0.1933 (11)	0.059 (3)	0.38 (3)
C14A	−0.2779 (13)	0.6523 (8)	−0.1247 (6)	0.034 (2)	0.62 (3)
F4A	−0.4522 (11)	0.6827 (10)	−0.1123 (11)	0.0398 (19)	0.62 (3)
F5A	−0.2538 (17)	0.5654 (8)	−0.1889 (9)	0.044 (2)	0.62 (3)
F6A	−0.1972 (17)	0.7326 (11)	−0.1815 (8)	0.053 (2)	0.62 (3)
O1	0.1106 (2)	0.31186 (14)	0.45777 (13)	0.0156 (4)	
O2	0.2954 (2)	0.44209 (14)	0.43387 (13)	0.0154 (4)	
O3	0.0284 (2)	0.60139 (14)	0.32975 (13)	0.0159 (4)	
O4	−0.1488 (2)	0.46706 (14)	0.35423 (13)	0.0160 (4)	
C2	0.6966 (4)	0.1020 (2)	0.3359 (2)	0.0258 (6)	
C3	0.5312 (4)	0.0680 (2)	0.3649 (2)	0.0287 (6)	
H3	0.5155	−0.0086	0.3645	0.034*	
C4	0.3887 (4)	0.1452 (2)	0.3945 (2)	0.0238 (6)	
H4	0.2752	0.1218	0.4143	0.029*	
C5	0.4124 (3)	0.2572 (2)	0.39516 (19)	0.0169 (5)	
C6	0.5781 (3)	0.2909 (2)	0.3642 (2)	0.0189 (5)	
H6	0.5937	0.3677	0.3635	0.023*	
C7	0.7206 (3)	0.2141 (2)	0.3345 (2)	0.0231 (6)	
H7	0.8337	0.2376	0.3133	0.028*	
C8	0.2638 (3)	0.3418 (2)	0.43068 (19)	0.0159 (5)	
C9	−0.0782 (3)	0.5447 (2)	0.2967 (2)	0.0173 (5)	
C10	−0.1245 (3)	0.5704 (2)	0.1862 (2)	0.0190 (5)	
C11	−0.2181 (3)	0.4987 (2)	0.1418 (2)	0.0233 (6)	
H11	−0.2507	0.4327	0.1815	0.028*	
C12	−0.2635 (4)	0.5235 (3)	0.0398 (2)	0.0284 (7)	
H12	−0.3263	0.4743	0.0091	0.034*	
C13	−0.2173 (4)	0.6201 (3)	−0.0171 (2)	0.0282 (7)	
C15	−0.1237 (4)	0.6919 (3)	0.0260 (2)	0.0304 (7)	
H15	−0.0920	0.7580	−0.0138	0.036*	
C16	−0.0768 (4)	0.6667 (2)	0.1275 (2)	0.0250 (6)	
H16	−0.0117	0.7153	0.1572	0.030*	
O1S	−0.3188 (2)	0.31076 (15)	0.58783 (14)	0.0215 (4)	0.397 (15)
C1S	−0.237 (3)	0.1999 (13)	0.6187 (15)	0.025 (3)	0.397 (15)
H1SA	−0.1085	0.1989	0.6102	0.030*	0.397 (15)
H1SB	−0.2658	0.1435	0.5728	0.030*	0.397 (15)
C2S	−0.310 (5)	0.175 (3)	0.735 (2)	0.032 (4)	0.397 (15)
H2SA	−0.2329	0.1961	0.7844	0.038*	0.397 (15)
H2SB	−0.3254	0.0946	0.7494	0.038*	0.397 (15)
C3S	−0.4855 (15)	0.2465 (9)	0.7481 (8)	0.033 (3)	0.397 (15)
H3SA	−0.5817	0.2005	0.7457	0.039*	0.397 (15)
H3SB	−0.5062	0.2823	0.8180	0.039*	0.397 (15)
C4S	−0.4781 (18)	0.3292 (12)	0.6608 (11)	0.020 (3)	0.397 (15)
H4SA	−0.5790	0.3272	0.6217	0.024*	0.397 (15)
H4SB	−0.4868	0.4040	0.6896	0.024*	0.397 (15)
O1T	−0.3188 (2)	0.31076 (15)	0.58783 (14)	0.0215 (4)	0.603 (15)
C1T	−0.2628 (19)	0.1954 (9)	0.6098 (9)	0.025 (2)	0.603 (15)
H1TA	−0.1365	0.1778	0.5831	0.030*	0.603 (15)
H1TB	−0.3303	0.1473	0.5756	0.030*	0.603 (15)
C2T	−0.298 (3)	0.1793 (19)	0.7318 (12)	0.029 (3)	0.603 (15)
H2TA	−0.1874	0.1726	0.7628	0.035*	0.603 (15)
H2TB	−0.3569	0.1113	0.7536	0.035*	0.603 (15)
C3T	−0.4179 (10)	0.2836 (6)	0.7699 (4)	0.0307 (17)	0.603 (15)
H3TA	−0.5269	0.2626	0.8142	0.037*	0.603 (15)
H3TB	−0.3577	0.3276	0.8133	0.037*	0.603 (15)
C4T	−0.4562 (12)	0.3465 (8)	0.6735 (7)	0.021 (2)	0.603 (15)
H4TA	−0.4587	0.4273	0.6826	0.025*	0.603 (15)
H4TB	−0.5717	0.3322	0.6568	0.025*	0.603 (15)
C5S	0.2278 (18)	0.8776 (12)	−0.0236 (14)	0.077 (4)	0.381 (5)
H5SA	0.2614	0.8639	−0.1002	0.116*	0.381 (5)
H5SB	0.0998	0.8845	−0.0058	0.116*	0.381 (5)
H5SC	0.2789	0.8154	0.0200	0.116*	0.381 (5)
C6S	0.2985 (18)	0.9909 (13)	0.0015 (11)	0.070 (3)	0.381 (5)
H6SA	0.2682	1.0516	−0.0519	0.084*	0.381 (5)
H6SB	0.2462	1.0141	0.0744	0.084*	0.381 (5)
C7S	0.5068 (17)	0.9639 (12)	−0.0061 (15)	0.075 (4)	0.381 (5)
H7SA	0.5375	0.8957	0.0386	0.090*	0.381 (5)
H7SB	0.5615	0.9530	−0.0816	0.090*	0.381 (5)
C8S	0.571 (2)	1.0696 (17)	0.038 (3)	0.090 (5)	0.381 (5)
H8SA	0.5284	1.0750	0.1161	0.108*	0.381 (5)
H8SB	0.5270	1.1391	−0.0007	0.108*	0.381 (5)
C9S	0.781 (2)	1.049 (2)	0.0156 (16)	0.108 (6)	0.381 (5)
H9SA	0.8179	1.0141	−0.0534	0.162*	0.381 (5)
H9SB	0.8235	0.9998	0.0736	0.162*	0.381 (5)
H9SC	0.8292	1.1204	0.0128	0.162*	0.381 (5)
O2S	0.443 (5)	0.928 (3)	−0.016 (5)	0.076 (6)	0.119 (5)
C10S	0.337 (5)	1.033 (3)	−0.030 (4)	0.079 (6)	0.119 (5)
H10A	0.2310	1.0377	0.0246	0.095*	0.119 (5)
H10B	0.2999	1.0399	−0.1026	0.095*	0.119 (5)
C11S	0.447 (5)	1.125 (2)	−0.019 (3)	0.074 (6)	0.119 (5)
H11A	0.3840	1.1782	0.0338	0.089*	0.119 (5)
H11B	0.4788	1.1652	−0.0887	0.089*	0.119 (5)
C12S	0.610 (7)	1.062 (4)	0.023 (7)	0.087 (7)	0.119 (5)
H12A	0.7167	1.0959	−0.0106	0.105*	0.119 (5)
H12B	0.5991	1.0646	0.1020	0.105*	0.119 (5)
C13S	0.617 (5)	0.950 (3)	−0.008 (4)	0.082 (7)	0.119 (5)
H13A	0.6922	0.9400	−0.0786	0.099*	0.119 (5)
H13B	0.6662	0.8971	0.0462	0.099*	0.119 (5)

### Atomic displacement parameters (Å^2^)

**Table:**

	*U*^11^	*U*^22^	*U*^33^	*U*^12^	*U*^13^	*U*^23^
Mo1	0.01239 (11)	0.01700 (12)	0.01261 (12)	−0.00162 (8)	−0.00291 (8)	−0.00050 (8)
C1	0.030 (3)	0.027 (3)	0.051 (3)	0.002 (2)	−0.003 (2)	−0.009 (2)
F1	0.044 (2)	0.036 (2)	0.104 (4)	0.0089 (15)	−0.002 (3)	−0.040 (3)
F2	0.058 (2)	0.075 (3)	0.064 (2)	0.0443 (19)	−0.0200 (19)	−0.0107 (19)
F3	0.035 (2)	0.0470 (17)	0.092 (3)	0.0125 (14)	0.0261 (19)	−0.0070 (18)
C1A	0.030 (8)	0.048 (8)	0.084 (9)	0.013 (6)	−0.003 (6)	−0.033 (7)
F1A	0.033 (7)	0.043 (6)	0.116 (13)	0.016 (5)	0.003 (9)	−0.029 (8)
F2A	0.028 (6)	0.049 (7)	0.101 (11)	0.012 (4)	−0.011 (6)	−0.029 (7)
F3A	0.042 (8)	0.092 (9)	0.095 (8)	0.030 (6)	0.010 (6)	−0.029 (6)
C14	0.035 (6)	0.070 (6)	0.021 (7)	0.018 (4)	−0.010 (4)	−0.003 (5)
F4	0.043 (5)	0.081 (7)	0.022 (4)	0.033 (5)	−0.009 (4)	0.006 (6)
F5	0.058 (7)	0.091 (5)	0.015 (5)	0.000 (4)	−0.008 (5)	−0.009 (4)
F6	0.054 (5)	0.099 (8)	0.017 (3)	0.008 (5)	−0.009 (3)	0.017 (5)
C14A	0.033 (4)	0.050 (4)	0.017 (4)	0.008 (3)	−0.007 (3)	−0.004 (3)
F4A	0.032 (2)	0.060 (4)	0.025 (3)	0.013 (2)	−0.013 (2)	0.000 (3)
F5A	0.048 (4)	0.064 (3)	0.016 (3)	0.022 (3)	−0.011 (3)	−0.014 (2)
F6A	0.061 (4)	0.075 (5)	0.026 (3)	−0.017 (3)	−0.020 (3)	0.021 (3)
O1	0.0144 (9)	0.0176 (9)	0.0153 (9)	−0.0026 (7)	−0.0035 (7)	0.0000 (7)
O2	0.0133 (8)	0.0179 (9)	0.0154 (9)	−0.0026 (7)	−0.0026 (7)	0.0001 (7)
O3	0.0141 (8)	0.0195 (9)	0.0146 (9)	−0.0032 (7)	−0.0033 (7)	−0.0001 (7)
O4	0.0144 (8)	0.0195 (9)	0.0149 (9)	−0.0025 (7)	−0.0040 (7)	−0.0005 (7)
C2	0.0243 (15)	0.0243 (15)	0.0277 (15)	0.0034 (12)	−0.0031 (12)	−0.0055 (12)
C3	0.0283 (15)	0.0207 (14)	0.0370 (17)	−0.0003 (12)	−0.0040 (13)	−0.0064 (12)
C4	0.0199 (14)	0.0250 (15)	0.0271 (15)	−0.0037 (11)	−0.0032 (11)	−0.0030 (11)
C5	0.0167 (13)	0.0216 (13)	0.0125 (12)	−0.0004 (10)	−0.0047 (10)	0.0005 (10)
C6	0.0183 (13)	0.0203 (13)	0.0185 (13)	−0.0005 (10)	−0.0058 (10)	−0.0001 (10)
C7	0.0155 (13)	0.0295 (15)	0.0240 (14)	−0.0001 (11)	−0.0032 (11)	−0.0030 (11)
C8	0.0171 (13)	0.0208 (14)	0.0105 (12)	−0.0029 (10)	−0.0051 (10)	0.0016 (10)
C9	0.0132 (12)	0.0203 (13)	0.0176 (13)	0.0018 (10)	−0.0014 (10)	−0.0023 (10)
C10	0.0159 (13)	0.0262 (14)	0.0136 (12)	0.0035 (10)	−0.0017 (10)	−0.0035 (10)
C11	0.0199 (13)	0.0299 (15)	0.0198 (14)	0.0007 (11)	−0.0039 (11)	−0.0031 (11)
C12	0.0217 (14)	0.0448 (18)	0.0206 (14)	0.0020 (13)	−0.0093 (11)	−0.0112 (13)
C13	0.0221 (14)	0.0449 (18)	0.0149 (13)	0.0080 (13)	−0.0029 (11)	−0.0025 (12)
C15	0.0299 (16)	0.0399 (18)	0.0187 (14)	0.0002 (13)	−0.0025 (12)	0.0068 (12)
C16	0.0246 (14)	0.0310 (15)	0.0195 (14)	−0.0032 (12)	−0.0045 (11)	0.0010 (11)
O1S	0.0183 (9)	0.0200 (9)	0.0255 (10)	−0.0013 (7)	−0.0011 (7)	−0.0022 (7)
C1S	0.021 (5)	0.016 (4)	0.037 (5)	−0.004 (4)	−0.001 (4)	−0.003 (3)
C2S	0.035 (6)	0.028 (6)	0.032 (5)	−0.003 (5)	−0.003 (4)	0.004 (4)
C3S	0.034 (4)	0.028 (4)	0.031 (4)	−0.002 (3)	0.009 (3)	0.002 (3)
C4S	0.010 (4)	0.028 (5)	0.024 (4)	−0.008 (3)	−0.006 (3)	−0.005 (3)
O1T	0.0183 (9)	0.0200 (9)	0.0255 (10)	−0.0013 (7)	−0.0011 (7)	−0.0022 (7)
C1T	0.020 (4)	0.021 (3)	0.033 (3)	−0.005 (2)	0.001 (3)	−0.002 (2)
C2T	0.031 (4)	0.022 (4)	0.033 (4)	0.002 (4)	−0.009 (3)	0.005 (3)
C3T	0.026 (3)	0.037 (3)	0.025 (2)	0.005 (3)	0.003 (2)	0.002 (2)
C4T	0.015 (3)	0.021 (3)	0.027 (3)	−0.005 (3)	0.003 (3)	−0.003 (2)
C5S	0.080 (8)	0.080 (8)	0.064 (8)	0.009 (6)	−0.005 (6)	0.015 (7)
C6S	0.082 (8)	0.075 (8)	0.042 (6)	0.015 (6)	−0.005 (6)	0.027 (5)
C7S	0.086 (8)	0.071 (8)	0.056 (6)	0.013 (7)	−0.002 (6)	0.025 (6)
C8S	0.101 (9)	0.083 (8)	0.076 (11)	0.012 (7)	−0.011 (7)	0.013 (7)
C9S	0.109 (10)	0.130 (15)	0.079 (11)	0.002 (9)	−0.019 (8)	0.015 (12)
O2S	0.087 (11)	0.079 (10)	0.049 (11)	0.025 (9)	−0.011 (10)	0.020 (9)
C10S	0.094 (12)	0.078 (11)	0.052 (11)	0.022 (10)	−0.005 (11)	0.026 (10)
C11S	0.092 (12)	0.067 (11)	0.051 (11)	0.026 (10)	−0.012 (10)	0.031 (10)
C12S	0.101 (13)	0.084 (11)	0.067 (12)	0.013 (11)	−0.008 (11)	0.019 (11)
C13S	0.087 (12)	0.080 (11)	0.072 (11)	0.014 (11)	−0.020 (11)	0.025 (10)

### Geometric parameters (Å, º)

**Table:**

Mo1—O1	2.0996 (17)	C1S—C2S	1.512 (13)
Mo1—O4	2.1030 (17)	C1S—H1SA	0.9900
Mo1—O2^i^	2.1076 (17)	C1S—H1SB	0.9900
Mo1—Mo1^i^	2.1098 (7)	C2S—C3S	1.519 (17)
Mo1—O3^i^	2.1204 (17)	C2S—H2SA	0.9900
Mo1—O1T	2.5422 (19)	C2S—H2SB	0.9900
Mo1—O1S	2.5422 (19)	C3S—C4S	1.430 (12)
C1—F3	1.304 (12)	C3S—H3SA	0.9900
C1—F1	1.345 (11)	C3S—H3SB	0.9900
C1—F2	1.349 (11)	C4S—H4SA	0.9900
C1—C2	1.493 (6)	C4S—H4SB	0.9900
C1A—F1A	1.21 (7)	O1T—C1T	1.431 (9)
C1A—F2A	1.38 (5)	O1T—C4T	1.447 (7)
C1A—F3A	1.40 (7)	C1T—C2T	1.518 (11)
C1A—C2	1.494 (18)	C1T—H1TA	0.9900
C14—F6	1.314 (14)	C1T—H1TB	0.9900
C14—F4	1.341 (13)	C2T—C3T	1.535 (13)
C14—F5	1.341 (14)	C2T—H2TA	0.9900
C14—C13	1.500 (13)	C2T—H2TB	0.9900
C14A—F6A	1.326 (8)	C3T—C4T	1.434 (8)
C14A—F4A	1.348 (9)	C3T—H3TA	0.9900
C14A—F5A	1.349 (8)	C3T—H3TB	0.9900
C14A—C13	1.509 (8)	C4T—H4TA	0.9900
O1—C8	1.275 (3)	C4T—H4TB	0.9900
O2—C8	1.271 (3)	C5S—C6S	1.605 (14)
O2—Mo1^i^	2.1076 (17)	C5S—H5SA	0.9800
O3—C9	1.269 (3)	C5S—H5SB	0.9800
O3—Mo1^i^	2.1204 (17)	C5S—H5SC	0.9800
O4—C9	1.270 (3)	C6S—C7S	1.602 (14)
C2—C3	1.384 (4)	C6S—H6SA	0.9900
C2—C7	1.389 (4)	C6S—H6SB	0.9900
C3—C4	1.382 (4)	C7S—C8S	1.590 (15)
C3—H3	0.9500	C7S—H7SA	0.9900
C4—C5	1.390 (4)	C7S—H7SB	0.9900
C4—H4	0.9500	C8S—C9S	1.607 (15)
C5—C6	1.387 (4)	C8S—H8SA	0.9900
C5—C8	1.485 (3)	C8S—H8SB	0.9900
C6—C7	1.381 (4)	C9S—H9SA	0.9800
C6—H6	0.9500	C9S—H9SB	0.9800
C7—H7	0.9500	C9S—H9SC	0.9800
C9—C10	1.490 (3)	O2S—C13S	1.425 (18)
C10—C16	1.391 (4)	O2S—C10S	1.442 (18)
C10—C11	1.392 (4)	C10S—C11S	1.505 (18)
C11—C12	1.385 (4)	C10S—H10A	0.9900
C11—H11	0.9500	C10S—H10B	0.9900
C12—C13	1.382 (4)	C11S—C12S	1.53 (2)
C12—H12	0.9500	C11S—H11A	0.9900
C13—C15	1.385 (4)	C11S—H11B	0.9900
C15—C16	1.383 (4)	C12S—C13S	1.436 (18)
C15—H15	0.9500	C12S—H12A	0.9900
C16—H16	0.9500	C12S—H12B	0.9900
O1S—C4S	1.428 (11)	C13S—H13A	0.9900
O1S—C1S	1.465 (12)	C13S—H13B	0.9900
			
O1—Mo1—O4	89.95 (7)	H1SA—C1S—H1SB	108.6
O1—Mo1—O2^i^	176.69 (6)	C1S—C2S—C3S	102.6 (12)
O4—Mo1—O2^i^	89.57 (7)	C1S—C2S—H2SA	111.3
O1—Mo1—Mo1^i^	93.20 (5)	C3S—C2S—H2SA	111.3
O4—Mo1—Mo1^i^	92.37 (5)	C1S—C2S—H2SB	111.3
O2^i^—Mo1—Mo1^i^	90.10 (5)	C3S—C2S—H2SB	111.3
O1—Mo1—O3^i^	88.41 (7)	H2SA—C2S—H2SB	109.2
O4—Mo1—O3^i^	176.47 (7)	C4S—C3S—C2S	106.9 (10)
O2^i^—Mo1—O3^i^	91.89 (6)	C4S—C3S—H3SA	110.3
Mo1^i^—Mo1—O3^i^	90.84 (5)	C2S—C3S—H3SA	110.3
O1—Mo1—O1T	96.83 (6)	C4S—C3S—H3SB	110.3
O4—Mo1—O1T	98.97 (6)	C2S—C3S—H3SB	110.3
O2^i^—Mo1—O1T	80.01 (6)	H3SA—C3S—H3SB	108.6
Mo1^i^—Mo1—O1T	164.83 (4)	O1S—C4S—C3S	111.1 (8)
O3^i^—Mo1—O1T	78.13 (6)	O1S—C4S—H4SA	109.4
O1—Mo1—O1S	96.83 (6)	C3S—C4S—H4SA	109.4
O4—Mo1—O1S	98.97 (6)	O1S—C4S—H4SB	109.4
O2^i^—Mo1—O1S	80.01 (6)	C3S—C4S—H4SB	109.4
Mo1^i^—Mo1—O1S	164.83 (4)	H4SA—C4S—H4SB	108.0
O3^i^—Mo1—O1S	78.13 (6)	C1T—O1T—C4T	108.1 (6)
F3—C1—F1	106.4 (6)	C1T—O1T—Mo1	118.0 (6)
F3—C1—F2	105.0 (7)	C4T—O1T—Mo1	120.2 (5)
F1—C1—F2	106.0 (8)	O1T—C1T—C2T	104.5 (9)
F3—C1—C2	114.9 (8)	O1T—C1T—H1TA	110.8
F1—C1—C2	112.5 (7)	C2T—C1T—H1TA	110.8
F2—C1—C2	111.3 (6)	O1T—C1T—H1TB	110.8
F1A—C1A—F2A	105 (4)	C2T—C1T—H1TB	110.8
F1A—C1A—F3A	117 (3)	H1TA—C1T—H1TB	108.9
F2A—C1A—F3A	97 (4)	C1T—C2T—C3T	105.4 (6)
F1A—C1A—C2	118 (5)	C1T—C2T—H2TA	110.7
F2A—C1A—C2	108 (3)	C3T—C2T—H2TA	110.7
F3A—C1A—C2	109 (4)	C1T—C2T—H2TB	110.7
F6—C14—F4	106.6 (13)	C3T—C2T—H2TB	110.7
F6—C14—F5	108.3 (13)	H2TA—C2T—H2TB	108.8
F4—C14—F5	106.3 (15)	C4T—C3T—C2T	105.6 (7)
F6—C14—C13	111.0 (12)	C4T—C3T—H3TA	110.6
F4—C14—C13	111.7 (14)	C2T—C3T—H3TA	110.6
F5—C14—C13	112.6 (14)	C4T—C3T—H3TB	110.6
F6A—C14A—F4A	108.9 (7)	C2T—C3T—H3TB	110.6
F6A—C14A—F5A	105.8 (7)	H3TA—C3T—H3TB	108.7
F4A—C14A—F5A	104.3 (8)	C3T—C4T—O1T	106.8 (5)
F6A—C14A—C13	114.5 (7)	C3T—C4T—H4TA	110.4
F4A—C14A—C13	110.8 (8)	O1T—C4T—H4TA	110.4
F5A—C14A—C13	112.0 (8)	C3T—C4T—H4TB	110.4
C8—O1—Mo1	115.86 (15)	O1T—C4T—H4TB	110.4
C8—O2—Mo1^i^	118.49 (15)	H4TA—C4T—H4TB	108.6
C9—O3—Mo1^i^	117.28 (15)	C6S—C5S—H5SA	109.5
C9—O4—Mo1	116.71 (15)	C6S—C5S—H5SB	109.5
C3—C2—C7	120.3 (3)	H5SA—C5S—H5SB	109.5
C3—C2—C1	121.5 (5)	C6S—C5S—H5SC	109.5
C7—C2—C1	118.0 (5)	H5SA—C5S—H5SC	109.5
C3—C2—C1A	116 (2)	H5SB—C5S—H5SC	109.5
C7—C2—C1A	124 (3)	C7S—C6S—C5S	106.0 (10)
C4—C3—C2	120.2 (3)	C7S—C6S—H6SA	110.5
C4—C3—H3	119.9	C5S—C6S—H6SA	110.5
C2—C3—H3	119.9	C7S—C6S—H6SB	110.5
C3—C4—C5	119.7 (3)	C5S—C6S—H6SB	110.5
C3—C4—H4	120.2	H6SA—C6S—H6SB	108.7
C5—C4—H4	120.2	C8S—C7S—C6S	105.2 (12)
C6—C5—C4	119.8 (2)	C8S—C7S—H7SA	110.7
C6—C5—C8	119.0 (2)	C6S—C7S—H7SA	110.7
C4—C5—C8	121.2 (2)	C8S—C7S—H7SB	110.7
C7—C6—C5	120.7 (2)	C6S—C7S—H7SB	110.7
C7—C6—H6	119.7	H7SA—C7S—H7SB	108.8
C5—C6—H6	119.7	C7S—C8S—C9S	104.7 (14)
C6—C7—C2	119.3 (3)	C7S—C8S—H8SA	110.8
C6—C7—H7	120.4	C9S—C8S—H8SA	110.8
C2—C7—H7	120.4	C7S—C8S—H8SB	110.8
O2—C8—O1	122.3 (2)	C9S—C8S—H8SB	110.8
O2—C8—C5	118.2 (2)	H8SA—C8S—H8SB	108.9
O1—C8—C5	119.5 (2)	C8S—C9S—H9SA	109.5
O3—C9—O4	122.8 (2)	C8S—C9S—H9SB	109.5
O3—C9—C10	119.1 (2)	H9SA—C9S—H9SB	109.5
O4—C9—C10	118.1 (2)	C8S—C9S—H9SC	109.5
C16—C10—C11	119.7 (2)	H9SA—C9S—H9SC	109.5
C16—C10—C9	120.3 (2)	H9SB—C9S—H9SC	109.5
C11—C10—C9	120.0 (2)	C13S—O2S—C10S	108.7 (18)
C12—C11—C10	120.0 (3)	O2S—C10S—C11S	107.7 (16)
C12—C11—H11	120.0	O2S—C10S—H10A	110.2
C10—C11—H11	120.0	C11S—C10S—H10A	110.2
C13—C12—C11	119.8 (3)	O2S—C10S—H10B	110.2
C13—C12—H12	120.1	C11S—C10S—H10B	110.2
C11—C12—H12	120.1	H10A—C10S—H10B	108.5
C12—C13—C15	120.8 (3)	C10S—C11S—C12S	103.5 (16)
C12—C13—C14	117.3 (7)	C10S—C11S—H11A	111.1
C15—C13—C14	121.9 (7)	C12S—C11S—H11A	111.1
C12—C13—C14A	120.1 (5)	C10S—C11S—H11B	111.1
C15—C13—C14A	119.0 (5)	C12S—C11S—H11B	111.1
C16—C15—C13	119.5 (3)	H11A—C11S—H11B	109.0
C16—C15—H15	120.2	C13S—C12S—C11S	105.2 (19)
C13—C15—H15	120.2	C13S—C12S—H12A	110.7
C15—C16—C10	120.3 (3)	C11S—C12S—H12A	110.7
C15—C16—H16	119.9	C13S—C12S—H12B	110.7
C10—C16—H16	119.9	C11S—C12S—H12B	110.7
C4S—O1S—C1S	106.0 (9)	H12A—C12S—H12B	108.8
C4S—O1S—Mo1	131.9 (7)	O2S—C13S—C12S	108 (2)
C1S—O1S—Mo1	110.8 (10)	O2S—C13S—H13A	110.1
O1S—C1S—C2S	107.0 (10)	C12S—C13S—H13A	110.1
O1S—C1S—H1SA	110.3	O2S—C13S—H13B	110.1
C2S—C1S—H1SA	110.3	C12S—C13S—H13B	110.1
O1S—C1S—H1SB	110.3	H13A—C13S—H13B	108.4
C2S—C1S—H1SB	110.3		
			
F3—C1—C2—C3	148.4 (6)	C11—C12—C13—C15	0.9 (4)
F1—C1—C2—C3	26.5 (11)	C11—C12—C13—C14	−179.0 (8)
F2—C1—C2—C3	−92.4 (8)	C11—C12—C13—C14A	−175.4 (5)
F3—C1—C2—C7	−36.4 (9)	F6—C14—C13—C12	−144.3 (13)
F1—C1—C2—C7	−158.3 (6)	F4—C14—C13—C12	96.8 (18)
F2—C1—C2—C7	82.8 (9)	F5—C14—C13—C12	−22.7 (17)
F1A—C1A—C2—C3	−20 (6)	F6—C14—C13—C15	35.7 (17)
F2A—C1A—C2—C3	−139 (3)	F4—C14—C13—C15	−83.1 (18)
F3A—C1A—C2—C3	117 (3)	F5—C14—C13—C15	157.3 (12)
F1A—C1A—C2—C7	164 (4)	F6A—C14A—C13—C12	−166.7 (8)
F2A—C1A—C2—C7	44 (6)	F4A—C14A—C13—C12	69.7 (10)
F3A—C1A—C2—C7	−60 (4)	F5A—C14A—C13—C12	−46.3 (11)
C7—C2—C3—C4	−1.1 (4)	F6A—C14A—C13—C15	16.9 (10)
C1—C2—C3—C4	174.0 (5)	F4A—C14A—C13—C15	−106.7 (9)
C1A—C2—C3—C4	−178 (3)	F5A—C14A—C13—C15	137.3 (8)
C2—C3—C4—C5	−0.1 (4)	C12—C13—C15—C16	−0.3 (4)
C3—C4—C5—C6	1.2 (4)	C14—C13—C15—C16	179.6 (8)
C3—C4—C5—C8	−177.5 (2)	C14A—C13—C15—C16	176.0 (5)
C4—C5—C6—C7	−1.1 (4)	C13—C15—C16—C10	−0.6 (4)
C8—C5—C6—C7	177.6 (2)	C11—C10—C16—C15	0.8 (4)
C5—C6—C7—C2	−0.1 (4)	C9—C10—C16—C15	−178.4 (2)
C3—C2—C7—C6	1.2 (4)	C4S—O1S—C1S—C2S	−23 (3)
C1—C2—C7—C6	−174.1 (5)	Mo1—O1S—C1S—C2S	125 (3)
C1A—C2—C7—C6	177 (3)	O1S—C1S—C2S—C3S	25 (4)
Mo1^i^—O2—C8—O1	1.0 (3)	C1S—C2S—C3S—C4S	−18 (4)
Mo1^i^—O2—C8—C5	−178.29 (15)	C1S—O1S—C4S—C3S	11.2 (18)
Mo1—O1—C8—O2	−1.3 (3)	Mo1—O1S—C4S—C3S	−127.7 (9)
Mo1—O1—C8—C5	177.94 (16)	C2S—C3S—C4S—O1S	5 (3)
C6—C5—C8—O2	−1.7 (3)	C4T—O1T—C1T—C2T	−28.3 (17)
C4—C5—C8—O2	177.0 (2)	Mo1—O1T—C1T—C2T	112.4 (15)
C6—C5—C8—O1	179.0 (2)	O1T—C1T—C2T—C3T	14 (2)
C4—C5—C8—O1	−2.3 (4)	C1T—C2T—C3T—C4T	5 (2)
Mo1^i^—O3—C9—O4	−0.1 (3)	C2T—C3T—C4T—O1T	−21.8 (15)
Mo1^i^—O3—C9—C10	179.30 (16)	C1T—O1T—C4T—C3T	32.4 (11)
Mo1—O4—C9—O3	1.7 (3)	Mo1—O1T—C4T—C3T	−107.3 (6)
Mo1—O4—C9—C10	−177.75 (16)	C5S—C6S—C7S—C8S	−170.7 (15)
O3—C9—C10—C16	−11.2 (4)	C6S—C7S—C8S—C9S	−172.8 (17)
O4—C9—C10—C16	168.3 (2)	C13S—O2S—C10S—C11S	−6 (6)
O3—C9—C10—C11	169.7 (2)	O2S—C10S—C11S—C12S	−10 (5)
O4—C9—C10—C11	−10.8 (3)	C10S—C11S—C12S—C13S	22 (6)
C16—C10—C11—C12	−0.2 (4)	C10S—O2S—C13S—C12S	21 (7)
C9—C10—C11—C12	179.0 (2)	C11S—C12S—C13S—O2S	−27 (7)
C10—C11—C12—C13	−0.7 (4)		

Symmetry code: (i) −*x*, −*y*+1, −*z*+1.

## References

[bb1] Boiadjiev, S. E. & Lightner, D. A. (1999). *J. Phys. Org. Chem.* **12**, 751–757.

[bb2] Bruker (2015). *APEX2* and *SAINT*. Bruker AXS Inc., Madison, Wisconsin, USA.

[bb3] Cotton, F. A., Daniels, L. M., Hillard, E. A. & Murillo, C. A. (2002). *Inorg. Chem.* **41**, 2466–2470. 10.1021/ic025508c 11978114

[bb4] Cotton, F. A., Extine, M. & Gage, L. D. (1978). *Inorg. Chem.* **17**, 172–176.

[bb5] Cotton, F. A., Mester, Z. C. & Webb, T. R. (1974). *Acta Cryst.* B**30**, 2768–2770.

[bb6] Cotton, F. A. & Nocera, D. G. (2000). *Acc. Chem. Res.* **33**, 483–490. 10.1021/ar980116o 10913237

[bb7] Cotton, F. A. & Norman, J. G. (1971). *J. Coord. Chem.* **1**, 161–171.

[bb8] Coulson, C. A. & Fischer, I. (1949). *Philos. Mag.* **40**, 386–393.

[bb10] D’Oria, E. & Novoa, J. J. (2008). *CrystEngComm*, **10**, 423–436.

[bb11] Engebretson, D. S., Graj, E. M., Leroi, G. E. & Nocera, D. G. (1999). *J. Am. Chem. Soc.* **121**, 868–869.

[bb12] Engebretson, D. S., Zaleski, J. M., Leroi, G. E. & Nocera, D. G. (1994). *Science*, **265**, 759–762. 10.1126/science.265.5173.759 17736272

[bb13] Groom, C. R., Bruno, I. J., Lightfoot, M. P. & Ward, S. C. (2016). *Acta Cryst.* B**72**, 171–179. 10.1107/S2052520616003954 PMC4822653 27048719

[bb14] Han, L.-J. (2011). *Acta Cryst.* E**67**, m1289–m1290. 10.1107/S1600536811033411 PMC3200930 22058882

[bb15] Heitler, W. & London, F. (1927). *Z. Phys.* **44**, 455–472.

[bb16] James, H. M. & Coolidge, A. S. (1933). *J. Chem. Phys.* **1**, 825–835.

[bb17] Kawahara, S., Tsuzuki, S. & Uchimaru, T. (2004). *J. Phys. Chem. A*, **108**, 6744–6749.

[bb18] Krause, L., Herbst-Irmer, R., Sheldrick, G. M. & Stalke, D. (2015). *J. Appl. Cryst.* **48**, 3–10. 10.1107/S1600576714022985 PMC4453166 26089746

[bb19] Lawton, D. & Mason, R. (1965). *J. Am. Chem. Soc.* **87**, 921–922.

[bb20] Lennard-Jones, J. E. (1929). *Trans. Faraday Soc.* **25**, 668–686.

[bb21] Lewis, G. N. (1916). *J. Am. Chem. Soc.* **38**, 762–785.

[bb22] Müller, P., Herbst-Irmer, R., Spek, A., Schneider, T. & Sawaya, M. (2006). *Crystal Structure Refinement: a Crystallographer’s Guide to SHELXL*, p. 16. Oxford University Press.

[bb23] Mulliken, R. S. (1932). *Phys. Rev.* **41**, 751–758.

[bb24] Pauling, L. (1928). *Chem. Rev.* **5**, 173–213.

[bb25] Pence, L. E., Weisgerber, A. M. & Maounis, F. A. (1999). *J. Chem. Educ.* **76**, 404–405.

[bb26] Rumble, J. R. (2021). *CRC Handbook of Chemistry and Physics*, 102nd ed. Boca Raton: CRC Press.

[bb27] Sheldrick, G. M. (2008). *Acta Cryst.* A**64**, 112–122. 10.1107/S0108767307043930 18156677

[bb28] Sheldrick, G. M. (2015*a*). *Acta Cryst.* C**71**, 3–8.

[bb29] Sheldrick, G. M. (2015*b*). *Acta Cryst.* A**71**, 3–8.

[bb30] Sluis, P. van der & Spek, A. L. (1990). *Acta Cryst.* A**46**, 194–201.

[bb31] Spek, A. L. (2020). *Acta Cryst.* E**76**, 1–11. 10.1107/S2056989019016244 PMC6944088 31921444

[bb32] Zheng, S.-L., Vande Velde, C. M. L., Messerschmidt, M., Volkov, A., Gembicky, M. & Coppens, P. (2008). *Chem. Eur. J.* **14**, 706–713. 10.1002/chem.200701037 17955556

